# A Parent-Mediated Telehealth Program for Children with Autism Spectrum Disorder: Promoting Parents’ Ability to Stimulate the Children’s Learning, Reduce Parenting Stress, and Boost Their Sense of Parenting Empowerment

**DOI:** 10.1007/s10803-022-05482-6

**Published:** 2022-03-02

**Authors:** Manuel Gentile, Linda Messineo, Dario La Guardia, Marco Arrigo, Giuseppe Città, Antonia Ayala, Gaspare Cusimano, Pio Martines, Giovanna Mendolia, Mario Allegra

**Affiliations:** 1 Istituto per le Tecnologie Didattiche, Consiglio Nazionale delle Ricerche, via Ugo la Malfa, 153, 90146 Palermo, Italy; 2 Azienda Sanitaria Provinciale di Trapani, Cittadella della Salute, viale della Provincia, 2, 91016 Erice Casa-Santa, Trapani Italy

**Keywords:** Telehealth, Autism spectrum disorder, Parent training, Parental empowerment, Parental stress

## Abstract

This study investigates the effectiveness of a 6-month parent-mediated early intervention telehealth program for children with an autism spectrum disorder. The participants comprised a total of 27 parents. The findings showed that participation in the program promotes parents’ empowerment and reduce parental stress and a general improvement in the parents’ ability to stimulate children’s learning. Moreover, the study reveals an effect of parents’ age in mediating the relationship among the parents’ ability, stress, and empowerment levels. These results suggest that an early intervention telehealth program may help parents become aware of how to benefit from interactions with their children, promote their empowerment, and reduce parenting stress.

## Introduction

Within the field of health care, the use of information and communication technologies (ICT)—in various ways—has become increasingly widespread. The introduction of ICT in health care has contributed to the conception of new terms referring to the different kinds of interventions. These interventions directly or indirectly benefit the people affected by short or long-term health problems or diseases. Among these interventions, “telehealth” has had considerable success. In the literature, “telehealth” is defined as the practice of continuously and/or remotely delivering health-related services through electronic technologies (Marchi et al., 2021)—treatment services for different diseases, training services for health professionals, exchange of health-related information services, online instruction services for interventionists, connection services for caregivers and remote experts (Symon, 2001; Neely et al., 2017).

Caregivers, especially parents, are one of the groups that has benefited the most from the introduction of telehealth in healthcare. Telehealth has come to benefit caregivers in the following ways: (a) learning to reflect on situations; (b) gaining confidence to try new resolution strategies; (c) learning to accept and deal with the condition of the people they are assisting more consciously (Wallisch et al., 2019).

As the use of telehealth in the care of people with autism spectrum disorder (ASD) is characterized by replacing or complementing the existing healthcare and training practices for autistic individuals and their caregivers, it has numerous advantages (Ingersoll & Berger, 2015). The research demonstrated the efficacy of using telehealth for providing various services to individuals with ASD and their families in different areas and fields of treatment (Boisvert et al., 2010; Knutsen et al., 2016; Lindgren et al., 2016). As has been highlighted in the critical review of the last decade, telehealth methods permit the provision of early intervention, communication and language therapy, and diagnostic assessment (Ellison et al., 2021). In a systematic review, the current trends in telehealth applications to deliver social communications interventions were investigated (Simacek et al., 2020).

The authors have found some of the studies that highlight the potential benefits of using parent-mediated telehealth interventions to improve social communication skills, such as language skills and conversational functions. Furthermore, Simacek et al. (2020) have shown that, in the use of telehealth for the healthcare of autistic subjects, the ability to combine synchronous and asynchronous telehealth sessions is crucial. On the one hand, synchronous telehealth enables one to set up “live” interventions within a home context. It allows a live connection between a “tele-provider” (clinician, professional, educator) and those who are the beneficiaries of the intervention (individuals with ASD, caregivers).

On the other hand, asynchronous telehealth allows the caregivers of individuals with ASD to let a “tele-provider” manage the collection and transmission of audio/video data, who can view the data at suitable times and respond to the caregivers’ needs in the best possible manner.

In the specific context of parent-mediated telehealth pathways, many studies have highlighted the effectiveness of telehealth as a tool to improve not only autistic individuals but also their parents (Solomon & Soares, 2020; Heitzman-Powell et al., 2014). For nearly 20 years, the role and involvement of parents have been officially and repeatedly recognized as a key component in the early treatment of ASD and in improving the quality of life of families of individuals with ASD (Council, 2001; Keen et al., 2010; Tonge et al., 2006).

There are studies that have implemented telemedicine to provide training to parents and improve their engagement. These studies have found the children of these parents to show acceptability and improvement in social communication skills (Bearss et al., 2017; Ingersoll & Berger, 2015; Ingersoll et al., 2016; Kuravackel et al., 2017; Pickard et al., 2016; Vismara et al., 2013, 2018; Wainer & Ingersoll, 2014). Over time, parent coaching and the monitoring, implementation, and evaluation of parent intervention have been accredited as the best evidence-based practice (Ferguson et al., 2018).

Various reviews have examined the use of telehealth in creating, providing, and managing educational pathways for caregivers and parents of children with ASD (Boisvert et al., 2010; Wainer & Ingersoll, 2014). Within this context, while some studies have investigated parental knowledge acquisition using telehealth (Peter et al., 2014), certain reviews have focused on the role of telehealth in parents’ learning and skill acquisition during therapeutic interventions (Hamad et al., 2010). Further, some studies that addressed both the issues (Pickard et al., 2016) found the parents engaging in educational and coaching pathways delivered through telehealth to show enhanced knowledge and intervention skills and be aware agents of therapeutic interventions (Parsons et al., 2017).

According to Baggett et al. (2009), this phenomenon occurs because telehealth allows the integration of the principles of instructional design and adult learning. The primary effect of this integration is the increase in parents’ understanding, management, and implementation of effective interventions for their children with ASD (Vismara et al., 2018). These interventions are more effective if the entire parent-mediated intervention and parent training pathways are therapist-assisted.

According to Ingersoll et al. (2016), therapist-assisted programs seem to be more effective than self-directed programs. Parents can improve in many ways. Their perception of their children with ASD increases positively because online interactive communication facilitates a close connection between parents and healthcare professionals (Hermaszewska & Sin, 2021) and enables parents to focus on important psychoeducation principles. Therefore, the educational use of telehealth pathways in parent-mediated interventions for individuals with ASD has an incremental effect on parental competencies (knowledge and intervention skills) and empowerment and decreases the effect on parental stress (Ingersoll et al., 2016; Hermaszewska & Sin, 2021; Kuhn & Carter, 2006).

Many studies have shown the importance of empowerment. Programs that focus on the parent-child relationship and involve parents learning new skills have been found to have the largest effects on child behavior and parental behavior and skills (Kaminski et al., 2008) and the greatest effects on reducing parental stress (Kuhn & Carter, 2006). In the specific case of empowerment, the educational dimension inherent in distance learning processes delivered via parent-mediated telehealth pathways has been explicitly highlighted by a few studies (Allegra et al., 2019; Ingersoll et al., 2016; Wallisch et al., 2019). While engaged in the learning experience, parents learn to reflect on (and discuss) the modalities and strategies of action that have helped them. They are more likely to discuss how they have found solutions to problematic situations involving both professionals and their peers. More aware of their own behavior and that of their children, they gain the necessary confidence to try out new coping strategies. They also gain a more positive perception of their family situation (Wallisch et al., 2019).

Regarding parental stress, teaching parents how to intervene in different stressful situations appropriately and conscientiously makes them aware of the tools that are required to deal with these situations and improves their psychological well-being. The contribution of telehealth as a coaching tool has taken on a central role in recent years (as suggested by Ingersoll et al. (2016)). A study compared the two approaches adopted for conducting a telehealth-based parent-mediated intervention for young children with ASD (one self-directed and one therapist-assisted). Here, the constant and more easily accessible support offered by the telehealth tool allowed to improve parental self-efficacy and reduce parental stress in the cases of both approaches (Ingersoll et al., 2016). Telehealth (in general) and telehealth-based and parent-mediated interventions in ASD (specifically) have multiple effects on parents. The various components are reciprocally connected and can potentially improve parents’ skills (intervention skills and knowledge) and level of empowerment and decrease their stress levels.

To the best of our knowledge, there are only a few studies that have investigated in detail how empowerment and stress are related to the improvement in parents’ skills in a telehealth context for children with ASD (Ingersoll et al., 2016). Thus, this study aims to examine this relationship based on the following research questions/hypotheses.

This study examines the effects of the ATHENA telehealth program on the parents of children with ASD (Allegra et al., 2019). ATHENA is a training-and-care telehealth program that provides therapist-assisted and parent-mediated interventions for children with ASD. It is a 6-month program designed to provide remote assistance for parent-mediated early intervention services. It started in the outpatient clinic and quickly transitioned to in the child’s home with both synchronous and asynchronous sessions as the intervention program progressed. Parent coaching and the implementation, monitoring, and evaluation of the parent-child-relationship play a key role. To be precise, it focuses on the changes in parents’ levels of empowerment, stress levels, and their ability to stimulate their children’s learning during treatment sessions. We hypothesize there to be an improvement in the parents’ teaching ability, a decrease in their stress levels, and an increase in their sense of empowerment during the 6-month program.

Further, we examine if and how the parents’ abilities may be related to their levels of stress and empowerment. In particular, the aim is to verify if the expected improvements in skills correspond to a decrease in stress and an increase in empowerment. Finally, to improve the educational model and make it more effective in various family conditions, we test if the parents’ age and the initial level of their children’s observed symptoms impact these relationships.

The rest of the paper is organized as follows. Section 'Methods' describes the measures used in this study, the study sample, and the intervention structure, both in terms of the services made available to parents and the scheduling of activities. Section 'Results' illustrates the results, first regarding the effects on the individual dimensions and then examining their relations. Finally, Section 'Discussion and Conclusion' reports the significant findings of the study.

## Methods

The ATHENA program is a 6-month program designed to provide remote assistance for parent-mediated early intervention services. Over this period, the parent moves from being a passive observer of the therapist-child session to becoming the child’s primary teaching partner while sessions with the therapist are phased out.

In this section, we present the measures employed, the digital environment designed to support the telehealth program, the sample description, and the intervention structure in terms of services and its time development.

### Measures

#### Autism Diagnostic Observation Schedule-2

The second edition of the Autism Diagnostic Observation Schedule (ADOS-2) (Lord et al., 2012) was used during the diagnostic evaluation. The ADOS-2 is a play and activity based semi-structured observation of social behaviors, communication, and restricted/repetitive behaviors. It consists of activities that elicit behaviors related to the diagnosis of ASD. It comprises five different modules, each of which is appropriate for a specific child based on their chronological age and expressive language level. Calibrated severity scores (Esler et al., 2015; Gotham et al., 2009) were used to measure ASD severity at the beginning and end of treatment. The ADOS calibrated severity scores measure ASD severity based on the ADOS raw totals, with relative independence from verbal level and age.

#### Autism Diagnostic Interview, Revised

The Autism Diagnostic Interview, Revised (ADI-R) (Lord et al., 1994) was used during the diagnostic evaluation. The new ADI-R algorithms for toddlers and young preschoolers were used (Kim & Lord, 2011). The ADI-R is a semi-structured parent or caregiver interview about developmental history and current functioning. It has 93 items. It focuses on three functional domains: (a) language/communication, (b) reciprocal social interactions, and (c) restricted, repetitive, and stereotyped behaviors and interests.

#### Vineland Adaptive Behavior Scale

The Vineland Adaptive Behavior Scale (VABS) (Sparrow et al., 2005) is an assessment tool used to diagnose and assess the special needs of individuals. It gathers information pertaining to adaptive behaviors in the areas of socialization, communication, daily living, and basic motor skills via semi-structured interviews conducted with the children’s parents or caregivers.

#### Parenting Stress Index, Fourth Edition

The level of perceived parental stress was assessed using the shortened form of the Parenting Stress Index, Fourth Edition (PSI-4) (Abidin, 2012). It is a test formulated to explore the identification of stressors in parent-child relationships. It measures the specific problematic characteristics of the relationship between children (aged between 1 month and 12 years) and their parents. These problematic characteristics can alter the normal development of children, resulting in them facing issues such as emotional and/or behavioral disorders, and parents may risk perceiving their role in a dysfunctional manner. The instrument is based on the assumption that parental stress can originate from multiple sources and results from a combination of personal characteristics and inherent features of the parental role.

The test provides information that can be used to develop specific interventions and evaluate the outcome of the treatments. Parents respond to the questions on a scale ranging from 1 (strongly disagree) to 5 (strongly agree). The shortened form of PSI-4 is composed of 36 items, which are divided into three subscales. The Parental Distress subscale (12 items) explores the level of distress that parents feel in relation to their specific parental role. The Parent-Child Dysfunctional Interaction subscale (12 items) is focused on the parents’ expectations of their interactions with their children. The Difficult Child subscale (12 items) addresses the fundamental characteristics of children’s behavior that make them easy or difficult to handle.

#### Family Empowerment Scale

The parents’ sense of empowerment was assessed by using the Family Empowerment Scale (FES) (Koren et al., 1992). FES is a widely self-administered survey that evaluates the parents’ sense of empowerment at three levels: family, service system, and community. It was developed to measure these components of empowerment in families that raise children with emotional, behavioral, or mental disabilities. It is a 34-item rating scale (1 = very untrue; 5 = very true). It is used in many contexts and instances of different childhood disabilities such as ASD (e.g. Weiss et al., 2013). A high score in each subscale reflects a considerable sense of empowerment in three areas. Before administering it, we translated the FES into Italian with the permission of the authors. We used only the family subscale of the survey because it was the type of empowerment that was the most likely to improve during the treatment program. It investigates the parents’ sense of self-efficacy in managing daily concerns regarding taking care of children with ASD and consists of 12 items.

#### Observation Tool

To evaluate the parents’ ability to stimulate their children’s learning during the recorded video sessions, an observational tool like the Early Start Denver Model (ESDM) Parent Fidelity Tool (Rogers & Dawson, 2010) was created.

Ten types of parental behaviors were selected, and these were to be scored by the therapists on a 10-point Likert scale. These ten types of behaviors were as follows:

Item 1: ability to manage children’s emotional states and activation levels.

Item 2: use of positive emotions.

Item 3: ability to motivate children.

Item 4: ability to create joint activities.

Item 5: sensitivity toward children’s communicative signals.

Item 6: ability to create different opportunities for children to practice their communication goals.

Item 7: use of language that is appropriate to the level of children.

Item 8: to possess a large repertoire of games.

Item 9: to offer frequent learning opportunities.

Item 10: to provide clear instructions.

**Fig. 1:**
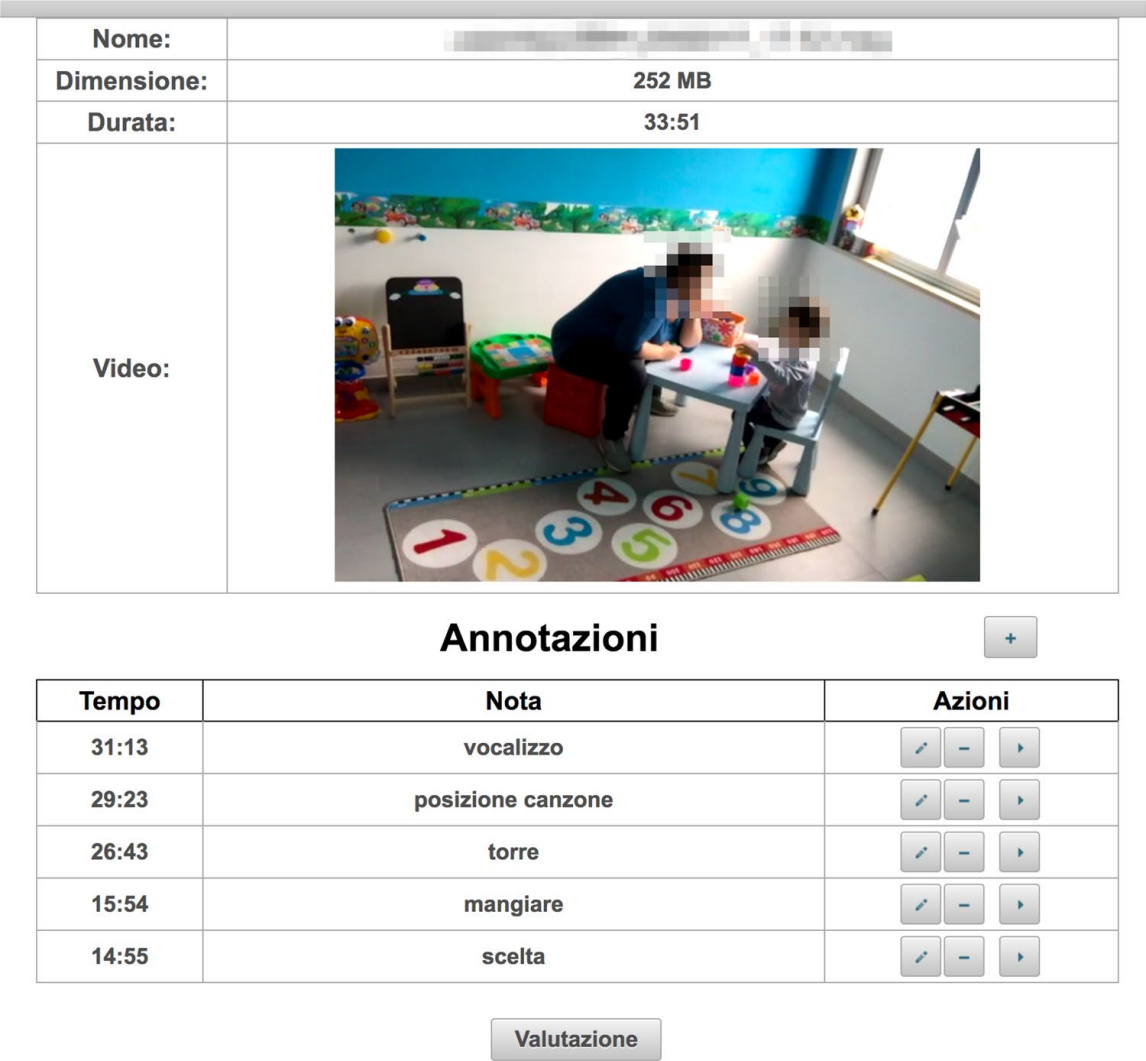
Web interface of the ATHENA video annotation tool

The therapists performed video evaluations through a dedicated web interface that allowed them to review and annotate the video sessions (Fig. 1). The annotation summary and the ability to directly access and review therapy highlights were designed to aid the assessment task.

### ATHENA Digital Environment

**Fig. 2:**
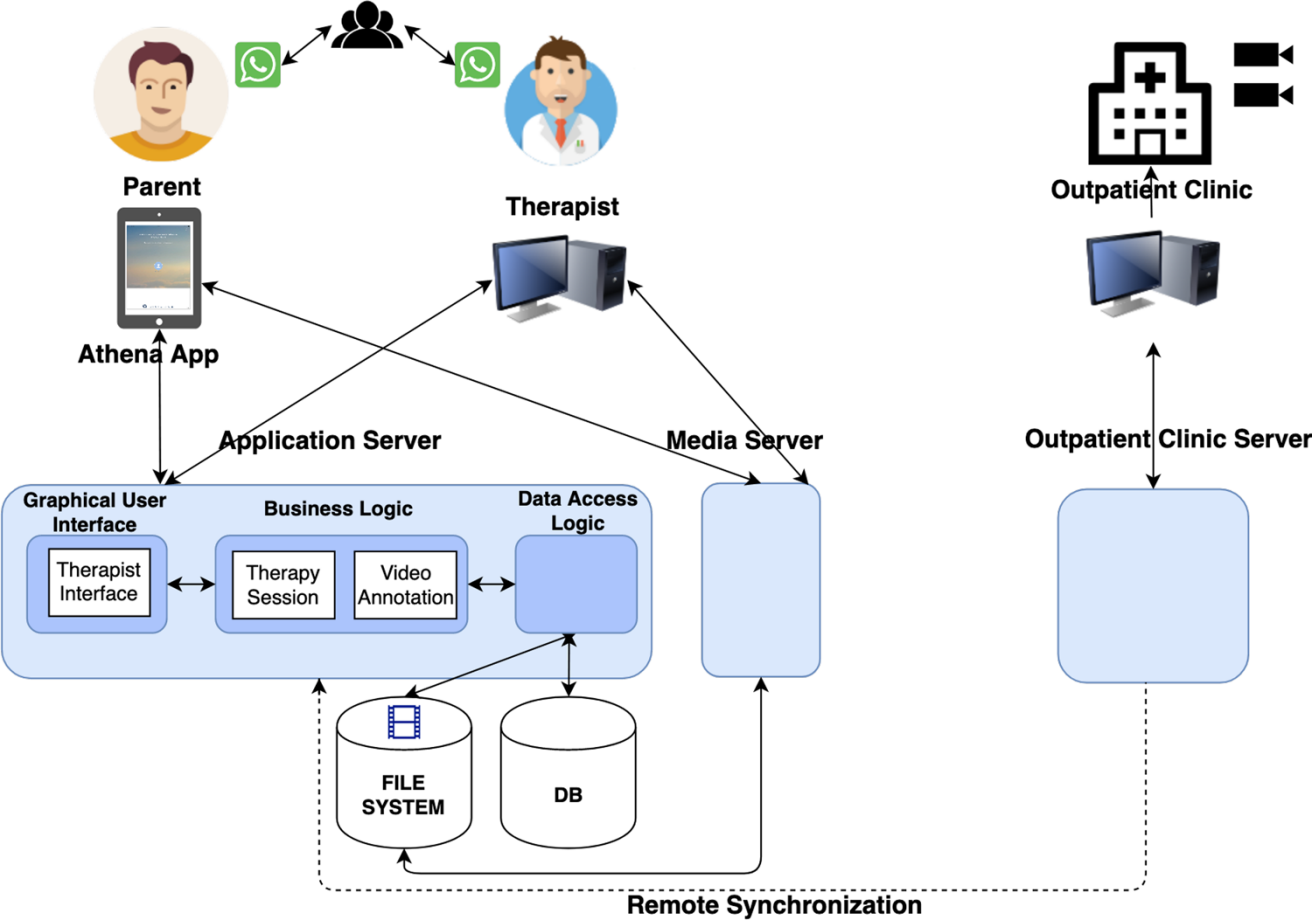
ATHENA digital environment

A telehealth delivery model meant to provide early intervention services from a distance was developed. Figure 2 presents the ATHENA digital environment. This architecture evolved during the planning phase of the intervention methodology to meet the following needs:

- provide telehealth services;
- monitor the parents’ activity and stimulate empowerment;
- record the therapists’ treatment sessions in the outpatient clinic;
- provide the therapists with the tools necessary to monitor the children’s progress.

The Application Server acts as the operating center where parents, therapists, health professionals, and all administrators can access ATHENA digital services but each with their own set of services and privileges. Two services were developed for parents: a telehealth system and a training system. An overview of successful telehealth systems (Chi & Demiris, 2015; Weinstein et al., 2014) has shown that many of these systems use mobile devices with simple, intuitive interfaces. Systems characterized by simplicity are preferred over those with many functions and customizing possibilities.

Based on these considerations, a mobile app named “ATHENA App” was created. It has a dashboard that opens with a simple click. ATHENA uses a simple “family-friendly” technology. The only instrument that the families needed to operate was a tablet, which was given to them at the beginning of the program. The families used the tablets to record videos and communicate with the therapists during the sessions.

#### ATHENA App

The ATHENA App was developed for tablets equipped with a Bluetooth headset to allow parents to interact in real-time with therapists as well as perform asynchronous activities. The decision to use a tablet was dictated by the need to provide families with a technologically friendly solution that could be used easily and simultaneously offers all the technical requirements necessary to conduct the training-and-care program. The tablet provided a simple and efficient audio-video system, allowing the parents to interact with therapists in synchronous sessions or record asynchronous sessions. Additionally, the possibility of using a mobile internet connection makes the system considerably flexible, easily transportable, adaptable to any house setting, and usable even by families who do not have an internet connection at home.

The system complies with privacy regulations. Any communication between the app and the server is conducted by a secure protocol (HTTPS), which is encrypted using an Secure Sockets Layer (SSL) certificate issued by the certification authority of the Gestione Ampliamento Rete Ricerca consortium (GARR) – Trans European Research and Education Networking Association (TERENA). Further, the audio/video communication is conducted through the Web Real-Time Communication (WebRTC) protocol.

**Fig. 3:**
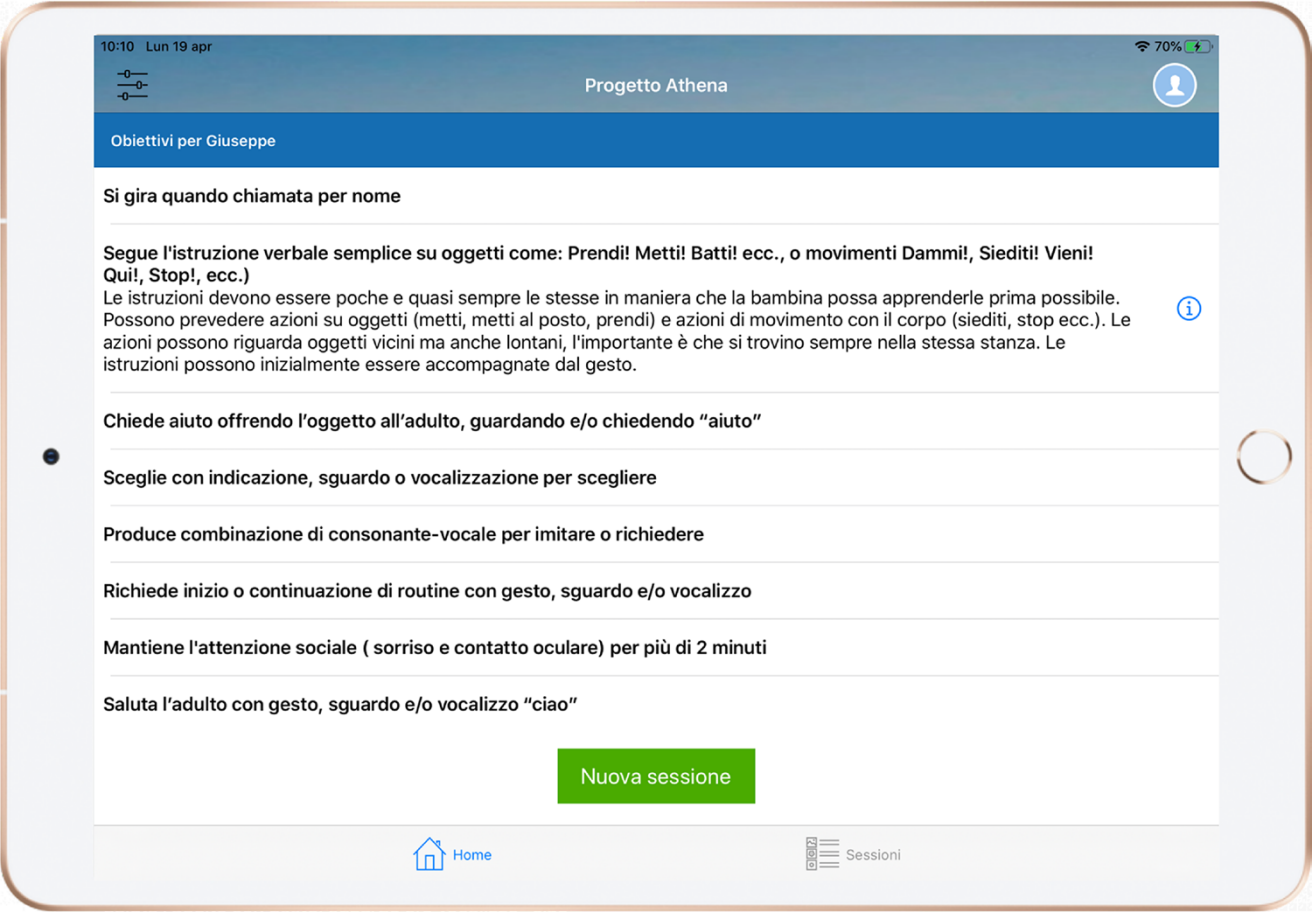
Dashboard view of ATHENA App

Once they are logged in, parents can access the activity control dashboard (Fig. 3). The app presents a list of objectives that the therapists have chosen for the parents to work on with their children. The dashboard was designed to be considerably intuitive via the proposal of a few buttons/choices to make the interaction and activation of functions easy and swift.

**Fig. 4:**
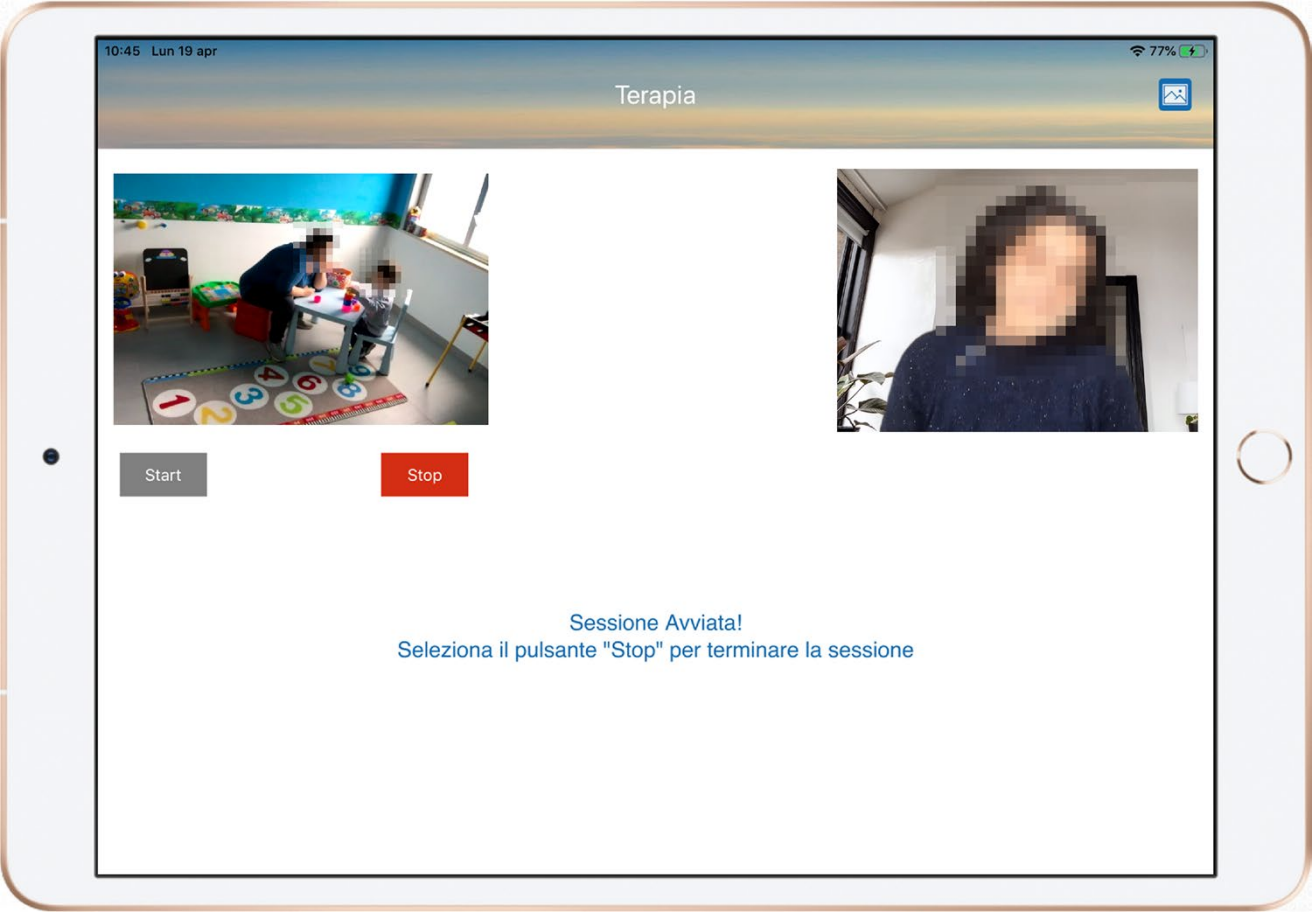
Example of a live session on ATHENA App

To activate a new session, parents simply had to touch the “New session” button. Subsequently, a connection with the ATHENA server will start, and a video audio acquisition interface will pop up with two panels concerning the video streams of the children and therapists, respectively (Fig. 4). Therapists’ video streams will be remotely activated by a professional only if they deem it appropriate and useful for the session. Parents can start the audio/video streaming of sessions by touching the “Start” button. The app also offers a “silent mode” function, which can be enabled by simply tapping the button at the top right of the interface. This darkens the tablet screen and displays only information about the elapsed time, avoiding all forms of distraction to children.

During the session, the app provides a two-way audio channel between the parents and therapists. Through the Bluetooth headset, parents can listen to their therapists’ suggestions silently, and therapists can listen to the environment audio and interactions of parents and their children. At the end of the session, a small 5-question questionnaire evaluating the activity performed is proposed. Once the questionnaire is sent, the app returns to the dashboard page. Parents can consult all the work sessions conducted and view the filled questionnaire and recorded video by tapping on the “Sessions” tab.

### Participants

The study participants comprised a total of 27 families of children with ASD or a pattern of development showing risks of developing ASD.

The sample was made up of 6 female and 21 male toddlers. Their ages ranged from 27 to 53 months. All children met the ADOS-2 cut-off for Autism Spectrum or Autism on Module 1 or a range of concerns for at least the mild/moderate criteria on the Toddler Module.

They also met the ADI-R toddler algorithm range of concern for at least the mild/moderate or satisfied criteria for an ASD classification on the normal algorithm. The mean mental age was 24.0 months ($SD=4.76$). The mean child age at intake was 36.0 months ($SD=6.41$).

Of the 27 selected families, 23 completed the entire intervention program. Thus, the analyses requiring a pre-post assessment were performed for the 23 families who completed the program. However, while evaluating the parental skill growth, the technique used (i.e., linear mixed models) allowed us to perform the analysis of the whole sample of enrolled families. The parents involved in the activities were mothers in 20 of the cases and fathers in three of the cases. The mean age of the parents was 35.78 years ($SD=7.17$).

### Procedure

**Fig. 5:**
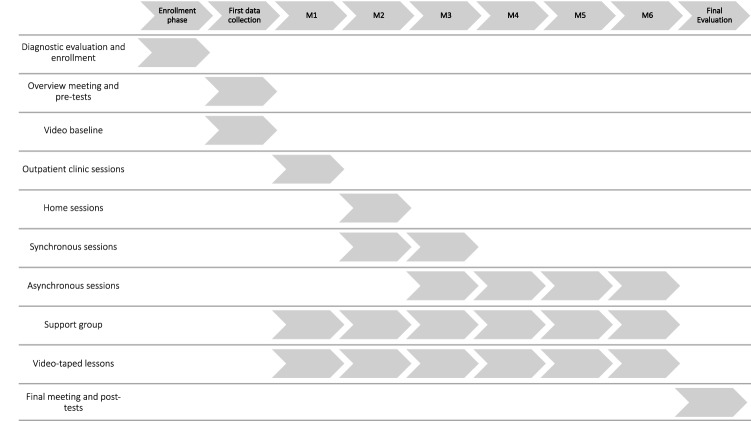
Phases and activities of the ATHENA program

The ATHENA program supports parents by offering synchronous and asynchronous counseling services through two systems: the telehealth system and the training system. Figure 5 presents the ATHENA intervention structure and shows how the different services are made available as the intervention progresses. Further, Table 1 illustrates the number and types of treatment sessions held per week.

**Table 1:** Calendar of the number and types of treatment sessions per week

Month	Outpatient clinic	Home	Synchronous	Asynchronous	Feedback
1	2				
2		1	1		
3			1	1	1
4				2	1
5				2	1
6				2	1

#### Diagnostic Evaluation and Enrollment

The first phase is the diagnostic evaluation aimed toward recruitment. Families were recruited from a public autism center in southern Italy. During the diagnostic evaluation, the second edition of the ADOS-2, the ADI-R, and the VABS were used.

The criteria for including families in the study program were positive results for ASD or possible ASD on both ADOS-2 and ADI-R and the mental age of the child being at least 18 months as evaluated by the VABS. The other criteria included the availability of one parent of each child for participation in all the activities and internet connection at home. Families with no internet connection were provided internet data connection for the entire duration of the treatment program by the research institute involved in the research study.

#### Overview Meeting and Pre-tests

Prior to the treatment’s start, the parents attended an overview meeting, which was immediately followed by an assessment session. During the first phase of the meeting, the complete treatment program was illustrated. The parents received a written document explaining the entire treatment program and their rights. They gave their written informed consent and then completed the measures, including the shortened form of the PSI-4 and the FES. Families were provided a tablet to allow them to record all the home therapies/training sessions with the therapists and participate in all three types of therapy sessions (synchronous, asynchronous, and feedback sessions). All the therapy/training sessions were recorded in the outpatient clinic as well as at home.

#### Video Baseline

Before starting the intervention program, parents recorded three 15–20 min videos of them interacting with their children in the natural home environment.

#### Video Lessons

The ATHENA program provided access to the video lessons on various topics of raising a child with ASD. This included selected chapters from the book “An Early Start for Your Child with Autism: Using Everyday Activities to Help Kids Connect, Communicate, and Learn” (Rogers et al., 2012)—a recommended reading material for the parents participating in the program.

#### Outpatient Clinic Sessions

During the first month, sessions were held twice a week for an hour in the outpatient clinic. The parents were present in the room as the therapists worked with the children. The focus of these first sessions was to assess the children’s abilities and develop their teaching goals. The therapy room had the necessary play materials for the sessions and a system for recording the videos on a local server (Outpatient Clinic Server). As the parents observed the sessions, they start learning how to interact with their children through the demonstrations. The recordings were stored in the local server and then synchronized with the central server using a remote synchronization link.

#### Home and Synchronous Sessions

After the first month, the intervention transitioned to consultative service, focusing on transferring knowledge and skills to the parents who could promote their children’s development and learning. The therapists provided the parents teaching objectives and strategies that they were expected to use with their children, as well as instructions to create daily play sessions. Teaching moments were encouraged during normal family activities such as mealtimes, bathing, and playing with siblings. In the second month, the therapists went to the homes of respective families once a week and worked directly with the children, continuing to model teaching strategies for the parents. When the “Synchronous” sessions were introduced, the therapist watched the ongoing sessions once a week through videoconferencing in real-time and offered suggestions to parents as they interacted with their children. Both types of sessions lasted for an hour.

#### Synchronous, Asynchronous and Feedback Sessions

In the third month of the project, synchronous and asynchronous sessions took place alternately. Synchronous therapies are sessions in which the therapists provide supervision to parents from a remote location to help them learn new strategies on how to teach their children. Asynchronous sessions are sessions in which the parents work alone with their children for 30 min and record the sessions. During the week, the therapists remotely watched the asynchronous video that the parents had previously recorded. By using the video annotation tools (Fig. 1), the therapist could take notes and highlight the behaviors and interactions of the child-parent pair. Subsequently, the therapist would give precise and detailed comments to the parents in the feedback sessions. Starting from the fourth month, the model included only asynchronous and feedback sessions.

#### Support Group

The ATHENA program offered additional support to parents in monthly group meetings with a psychotherapist—an expert in ASD—to discuss the strategies and principles presented in the video lessons, the concerns about their children’s progress in the project, or any other issue concerning their children on which they would like help. Both the parents—not just the parent participating in the program activities—were invited to participate. Additional help was provided via the possibility to discuss different topics with an ASD expert using the chat option provided by the messaging service.

#### Final Evaluation

ADOS-2 and VABS were repeated at the end of the 6-month period. In this period, the parents attended an exit session, during which they provided feedback about the program and completed the same measures administered at the beginning—PSI-4 and FES.

### Statistical Analysis

All the statistical analyses were performed using the version 3.6.1 of the R software (R Core Team, 2018). We estimated these models using the lme4 package (version 1.1.21) (Bates et al., 2015). Further, we described in detail how we performed the analysis of the three main aspects. First, we analyzed the effect of the ATHENA program on the ASD symptoms observed, the parents’ sense of empowerment in their families, and the parents’ stress. Subsequently, we analyzed the trend in the parents’ performance during the ATHENA program. Finally, we analyzed the interaction between the expected improvement in parents’ skills and the possible effect on stress and empowerment.

#### Single Dimension Analysys

To analyze the effect of the ATHENA program, three paired-samples *t* tests were conducted to compare (1) the number of ASD symptoms observed (which was measured by ADOS-2), (2) the parents’ sense of empowerment in their families measured by FES, and (3) the sum of parents’ stress (ST) measured by PSI-4, both before and after the intervention.

#### Evaluation of Videos

The videos were analyzed to prove the positive effect of the intervention on the parents’ ability to stimulate their children’s learning. The measure used is the observation tool described above. The observations of the outcome variables considered in this analysis are not independent as we repeated the measurements on the same subject. To deal with this condition, we relied on mixed linear models, which allowed us to reproduce the nested model and define the specific variance term for the intra-subject analysis. Let $Q_{p}$ be the level of quality observed by the therapist for parent *p*. The *step* variable is calculated as the difference in days between the day of the therapy linked to the observation and that of the first therapy session. First, we wanted to verify the effect of the *step* on the quality of the parent intervention observed by the therapist. To this end, we compared the following models:

1 $$\begin{aligned} Q_{p}= & {} \beta _{0}+ \beta _{0(p)}+ e_{p} \end{aligned}$$

2 $$\begin{aligned} Q_{p}= & {} \beta _{1}*item+\beta _{0(p)}+ e_{p} \end{aligned}$$

3 $$\begin{aligned} Q_{p}= & {} \beta _{1}*item+\beta _{2}*step+ \beta _{0(p)}+ e_{p} \end{aligned}$$

4 $$\begin{aligned} Q_{p}= & {} \beta _{1}*item+\beta _{2}*step+ \beta _{0(p)}+ \beta _{1(p)}*step+ e_{p} \end{aligned}$$

Model 1 is a classical random intercept used with a fixed mean model. In Model 2, the variable *item* is added to Model 1 to verify the fixed effect of the different items of the observation tool. The aim of Model 3 is to consider the fixed effect of the *step* variable. Finally, Model 4—a correlated random intercept and slope model—is added to verify the random effect of the *step* variable. Multiple fit indices were considered to compare models such as Akaike information criterion (AIC), Bayesian information criterion (BIC), and chi-square ($\chi ^2$).

#### Analysis on the Interaction Among the Different Dimension

One of the principal aims of this work has been to verify the relationship between the parents’ ability to stimulate their children’s learning and some of the measures generally used as a signal of the difficulties encountered by parents, such as the levels of stress and empowerment.

In particular, our goal is to verify if there is a relationship between the expected improvement in the parents’ skills induced by the training intervention on the one hand and the decrease in stress and boosting of empowerment on the other hand.

To this end, we first performed a simple correlation analysis between the $\Delta$ of the variables. The next step was to test if the variables such as the parents’ age or the initial level of the children’s observed symptoms played a role in the relationship between the improvement in parents’ ability and that of their stress and empowerment levels.

To this end, we conducted the analysis of the linear models described by Eqs. 5 and 6.

5 $$\begin{aligned} \Delta Total Stress = \Delta Ability * ParentAge + \Delta Ability * InitialAutismSymptoms \end{aligned}$$

6 $$\begin{aligned} \Delta Family Emp = \Delta Ability * ParentAge + \Delta Ability * InitialAutismSymptoms \end{aligned}$$

## Results

### Single Dimension Analysys

**Fig. 6:**
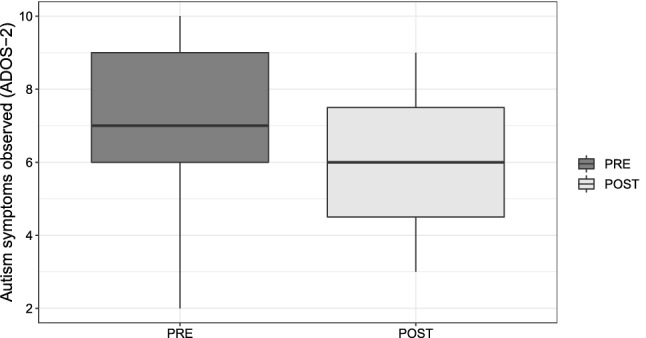
Distribution of the quantity of ASD symptoms observed before and after the ATHENA intervention

The paired-samples’ *t* test was conducted to compare the quantity of ASD symptoms observed, which was measured using ADOS-2 calibrated severity scores before ($M=7.260$, $SD=2.027$) and after the therapy ($M=6.040$, $SD=1.827$). Further, $t(22)=-2.74$, $p=0.006$, $d=0.630$ suggest a decrease in the symptoms observed. Thus, based on the quantity of ASD symptoms, the therapy is observed to have a medium positive effect on children (Fig. 6).

**Fig. 7:**
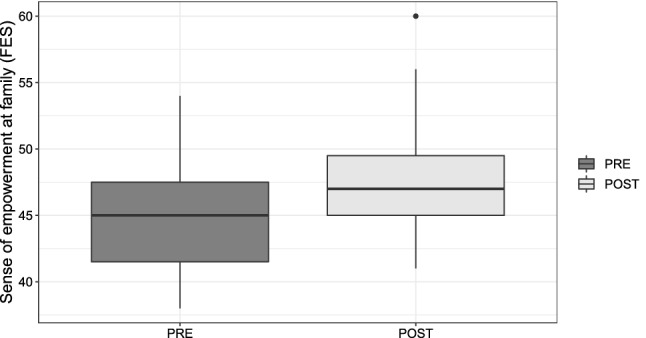
Sense of empowerment in family distribution before and after the ATHENA intervention

Similarly, the paired-samples *t* test was conducted to compare the parents’ sense of empowerment in families measured by FES before ($M = 45.0$, $SD = 4.09$) and after therapy ($M = 47.826$, $SD = 4.745$). Here, $t(22) = 2.25$, $p = 0.017$, $d = 0.638$ indicate an increased sense of empowerment; thus, the intervention had a medium positive effect on the parents’ sense of empowerment (Fig. 7).

**Fig. 8:**
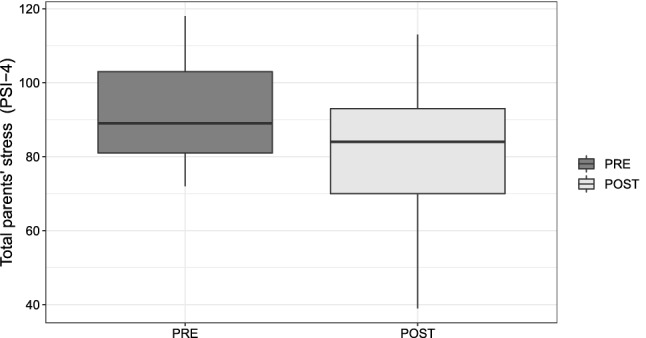
Distribution of the sum of parents’ stress before and after the ATHENA intervention

Finally, the paired-samples *t* test was conducted to compare the sum of parents’ stress (ST), which was measured by PSI-4 before ($M = 92.348$, $SD = 13.58$) and after the therapy ($M = 81.0$, $SD = 18.703$). Here, $t(22) = -4.16$, $p = 0.0002$, $d = 0.655$ indicate a decrease in stress levels; thus, the therapy had a discrete positive effect on the sum of parents’ stress (Fig. 8).

### Evaluation of Videos

While Table 2 presents the regression coefficients of the linear mixed models, Table 3 presents the ANOVA comparison of these models.

The analysis of the models highlights a significant fixed effect of the *item* and *step* variables. Model 4 accounts for the individual differences in the parental improvement process and presents the best fit for the data while confirming the significance of the *item* and *step* variables’ fixed effects. In particular, the coefficient of the *step* variable $$\beta _{2}=0.0105$$ ($$p = 0.0001 < 0.001^{****}$$) is positive, which indicates a significant improvement in the parents’ ability to stimulate their children’s learning over time.

The analysis of the overall effect on the individual parent of the *step* variable, obtained by adding the random effects to the fixed effect, shows that parents generally recorded the improvement in their ability. A slight worsening was noted only in four cases.

**Table 2:** Fits of linear mixed models

	Model 1	Model 2	Model 3	Model 4
(Intercept)	$$6.91^{***}$$			
	(0.13)			
Item $$1^{\hbox {a}}$$		$$6.66^{***}$$	$$6.07^{***}$$	$$6.11^{***}$$
		(0.14)	(0.15)	(0.20)
Item 2		$$7.09^{***}$$	$$6.49^{***}$$	$$6.54^{***}$$
		(0.14)	(0.15)	(0.20)
Item 3		$$6.80^{***}$$	$$6.20^{***}$$	$$6.25^{***}$$
		(0.14)	(0.15)	(0.20)
Item 4		$$7.19^{***}$$	$$6.59^{***}$$	$$6.64^{***}$$
		(0.14)	(0.15)	(0.20)
Item 5		$$6.95^{***}$$	$$6.36^{***}$$	$$6.40^{***}$$
		(0.14)	(0.15)	(0.20)
Item 6		$$6.91^{***}$$	$$6.31^{***}$$	$$6.36^{***}$$
		(0.14)	(0.15)	(0.20)
Item 7		$$7.10^{***}$$	$$6.50^{***}$$	$$6.55^{***}$$
		(0.14)	(0.15)	(0.20)
Item 8		$$6.66^{***}$$	$$6.06^{***}$$	$$6.11^{***}$$
		(0.14)	(0.15)	(0.20)
Item 9		$$6.83^{***}$$	$$6.23^{***}$$	$$6.28^{***}$$
		(0.14)	(0.15)	(0.20)
Item 10		$$6.92^{***}$$	$$6.33^{***}$$	$$6.37^{***}$$
		(0.14)	(0.15)	(0.20)
step			$$0.01^{***}$$	$$0.01^{***}$$
			(0.00)	(0.00)
AIC$^{\hbox {b}}$	14406.43	14384.86	14124.73	13925.91
BIC$^{\hbox {c}}$	14425.46	14461.00	14207.22	14021.08
Log likelihood	$-7200.21$	$-7180.43$	$-7049.37$	$-6947.95$
No. of obs.	4210	4210	4210	4210
No. of groups: id	23	23	23	23
Var: id (intercept)	0.35	0.35	0.38	0.76
Var: residual	1.76	1.73	1.62	1.52
Var: id step				0.00
Cov: id (intercept) step				$-0.01$

$$^{***}p<0.001$$; $$^{**}p<0.01$$; $$^{*}p<0.05$$

^a^The items are described in Sect. 'Observation Tool'

^b^Akaike information criterion

^c^Bayesian information criterion

**Table 3:** ANOVA comparison of the linear mixed models

	npar	AIC$^{\hbox {a}}$	BIC$^{\hbox {b}}$	Log likelihood	Deviance	Chisq	Df	Pr(>Chisq)
Model1	3.00	14404.10	14423.13	− 7199.05	14398.10			
Model2	12.00	14351.91	14428.05	− 7163.96	14327.91	70.19	9	0.0000
Model3	13.00	14078.45	14160.94	− 7026.23	14052.45	275.46	1	0.0000
Model4	15.00	13881.34	13976.52	− 6925.67	13851.34	201.11	2	0.0000

^a^Akaike information criterion

^b^Bayesian information criterion

### Analysis on the Interaction Found Among the Different Dimension

The correlation analysis did not reveal a statistically significant correlation between improvement in parents’ ability and total stress variation $\beta =-0.1896$; $t(21)=-0.89$ ($p = 0.4$). Also between improvement in parents’ ability and family empowerment variation, the correlation analysis did not reveal a statistically significant correlation $\beta =-0.1063$; $t(21)=-0.49$ ($p = 0.6$).

**Fig. 9:**
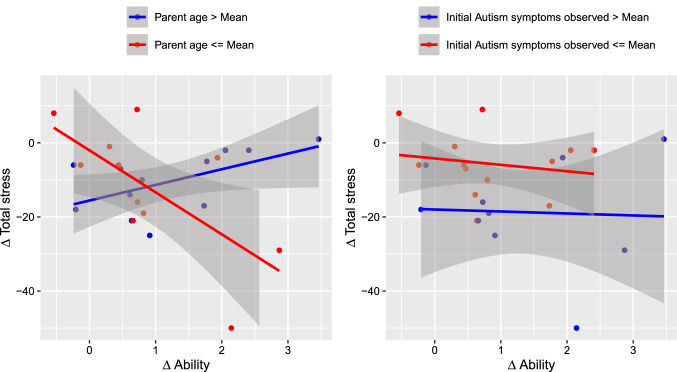
$\Delta$Total Stress vs $\Delta$Ability of the parents in relation to their age and the initial ASD symptoms observed

**Fig. 10:**
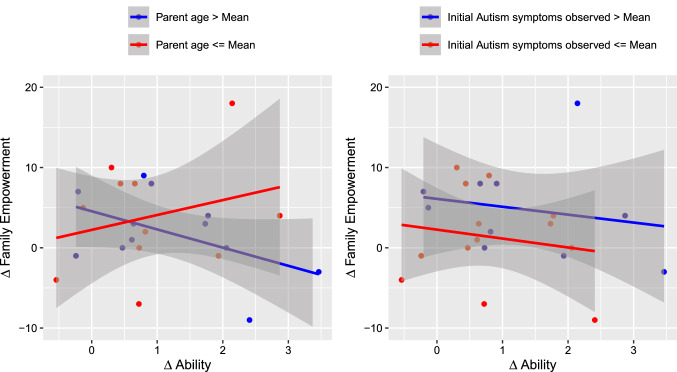
$\Delta$Family Empowerment vs $\Delta$Ability of the parents in relation to their age and the initial ASD symptoms observed

Nevertheless, Figs. 9 and 10 show that the parents’ age seemed to have impacted those relationships. This evidence has been confirmed by the results of the linear models described by equations 5 and 6 (see Table 4).

In particular, Model 5 (estimated using ordinary least squares) explained the significant and substantial proportion of the variance observed ($R^2 = 0.60$, $F(5, 17) = 5.04$, $p = 0.005$, $adj.R^2 = 0.48$). Within this model, the effect of $\Delta Ability$ was significantly negative ($\beta = -42.58, p < 0.01$), the effect of parent age was significantly negative ($\beta = -1.28, p < 0.01$), and the interaction effect of age on $\Delta Ability$ was significantly positive ($\beta = 1.34, p < 0.001$).

Finally, Model 6 (estimated using OLS) explained the not significant and substantial proportion of variance ($R^2 = 0.33$, $F(5, 17) = 1.69$, $p = 0.191$, $adj.R^2 = 0.14$). Within this model, only the interaction effect of age on $\Delta Ability$ was significantly negative ($\beta = -0.46, p < 0.05$).

**Table 4:** Fits of linear models

	Model 5	Model 6
(Intercept)	$41.30^{*}$	$-14.54$
	(19.05)	(11.30)
$$\Delta$$Ability	$$-42.58^{**}$$	12.39
	(12.21)	(7.25)
Parent age	$$-1.28^{**}$$	0.46
	(0.42)	(0.25)
Initial autism symptoms	$-0.44$	0.16
	(1.49)	(0.88)
$$\Delta$$Ability:parent age	$$1.34^{***}$$	$$-0.46^{*}$$
	(0.29)	(0.17)
$\Delta$Ability:initial autism symptoms	$-1.38$	0.59
	(1.25)	(0.74)
R$^2$	0.60	0.33
Adj. R$^2$	0.48	0.14
No. of obs.	23	23

$$^{***}p<0.001$$; $$^{**}p<0.01$$; $$^{*}p<0.05$$

## Discussion and Conclusion

This study aimed to investigate the effectiveness of the training-and-care telehealth program called ATHENA in promoting parents’ ability to stimulate their children’s learning, reduce parenting stress, and improve their sense of parenting empowerment.

The ATHENA program was based on a naturalistic developmental behavioral intervention. It involved a total of 27 parents of children with ASD. It was conducted over 6 months.

The principal aim of the program was to help parents become more active in their teaching role with their children via the provision of parent training, psychological support, and coaching. It took place primarily in the homes of children via the use of interactive social contexts such as play and daily routines.

Overall, the results of this study show a significant improvement in the quality of parental intervention as judged by the therapist, an improvement in parents’ sense of empowerment, and a reduction in parental stress.

The improvement in the parents’ abilities to stimulate their children’s learning was measured via the therapist’s observation of the videos of the parents’ asynchronous sessions. The video analysis supported the positive effect of the program intervention on the parents’ behavior while managing the interaction sessions with their children.

Furthermore, this study showed an increase in empowerment. In particular, the statistical analysis showed the intervention to have a medium positive effect on the parents’ sense of empowerment.

Finally, following the parents’ participation in the ATHENA program, we observed that the parents reported a reduction in the parental stress related to managing the daily concerns of raising children with ASD. A positive effect of the intervention program on the sum of parents’ stress, measured by the PSI-4, was estimated. This result contributes to the debate in the literature about the efficacy of parent-mediated interventions on parental stress reduction. There have been studies on non-ASD clinical populations that have found a positive association between parental interventions and reduced stress and an increased locus of control (Moreland et al., 2016).

A recent study demonstrated that the participation of parents in a parent training program positively affected both the child outcomes—such as disruptive behaviors—and parental factors such as parental competence and reduction in parental stress (Iadarola et al., 2018).

The positive results of this study may be ascribed to the benefit provided by the telehealth approaches in implementing a naturalistic developmental approach.

The parents introduced the intervention through play and everyday family routines, which affected the improvement of child outcomes as well as their abilities as co-therapists. These results are in agreement with previous research (Kaminski et al., 2008). As reported in a meta-analytic review of the aspects associated with parent efficacy, programs that focus on the inter parent-child interaction and involve parents learning new skills with their children have been shown to have a significant effect on both child behavior and parenting skills (Kaminski et al., 2008).

Regarding the relationship between the improvement in parenting skills and its effects on stress and empowerment levels, the data call for the need to have further investigation. Indeed, while it is not possible to present a significant correlation between these variations, further analysis conducted on the role of parental age and the initial level of the observed symptoms has brought out the effect of parental age. In particular, data have shown that the initial hypothesis of the positive effect of skill enhancement on the levels of stress and empowerment has been verified among younger parents. Conversely, in older parents, an opposite effect has been witnessed.

While it is entirely plausible that younger people may benefit from these educational approaches, perhaps because they are more open to using technology, the older parents’ negative effect is difficult to explain (AARP, 2016).

Further studies are needed to investigate this phenomenon in greater depth. They should (a) increase the sample size and (b) introduce new measures, such as technology acceptance, within the theoretical framework of the Unified Theory of Acceptance and Use of Technology, which could consistently explain how age is a moderating variable for the behavioral intention to use technology such as Venkatesh et al. (2003).

The strength of this study lies in the use of telehealth to provide the intervention program. Teaching parents in individual encounters, such as for the first month at the outpatient clinic, 2 months at home, and 3 months from a distance (synchronously and asynchronously), led to an improvement in their empowerment, a reduction in their stress levels, and significant positive effects on their social behaviors with their children. This study confirms the literature results pertaining to the effectiveness of the use of telemedicine as a service delivery method for ASD (Knutsen et al., 2016).

Telehealth programs can reduce costs and increase the availability of services, which is especially important in geographical areas that experience economic stress and/or lack quality services for ASD. The results have brought out the possible importance of providing a naturalistic developmental intervention to support the development of children and parents in their parenting roles.

## Limitations

This study has a few limitations. First, the study lacks a control group. Further research should consider the estimation of the differences in outcomes between a group of parents who have their children exposed to a parent-mediated intervention program and an equivalent parent group exposed to traditional children intervention. Another limitation of this study is the use of a relatively small sample of parents and their children. Future research should recruit larger samples of parents and their children to gain insights into the wider context.

## References

[CR1] AARP (2016) Caregivers & technology: What they want and need. Tech. rep., American Association of Retired Persons (AARP). 10.26419/res.00191.002

[CR2] Abidin R (2012). Parenting Stress Index-Fourth Edition (PSI-4).

[CR3] Allegra, M., Arrigo, M., Ayala, A., Cusimano, G., Gentile, M., La Guardia, D., Martines, P., Mendolia, G., & Messineo, L. (2019). Treatment and tele-rehabilitation home-based for Autistic spectrum disorder: The ATHENA project. *Ricerche di Psicologia, 42*(3), 505–518. 10.3280/RIP2019-003004

[CR4] Baggett KM, Davis B, Feil EG, Sheeber LL, Landry SH, Carta JJ, Leve C (2009). Technologies for expanding the reach of evidence-based interventions: Preliminary results for promoting social-emotional development in early childhood. Topics in Early Childhood Special Education.

[CR5] Bates D, Mächler M, Bolker B, Walker S (2015). Fitting linear mixed-effects models using lme4. Journal of Statistical Software.

[CR6] Bearss K, Burrell TL, Challa SA, Postorino V, Gillespie SE, Crooks C, Scahill L (2017). Feasibility of parent training via telehealth for children with autism spectrum disorder and disruptive behavior: A demonstration pilot. Journal of Autism and Developmental Disorders.

[CR7] Boisvert M, Lang R, Andrianopoulos M, Boscardin ML (2010). Telepractice in the assessment and treatment of individuals with autism spectrum disorders: A systematic review. Developmental Neurorehabilitation.

[CR8] Chi NC, Demiris G (2015). A systematic review of telehealth tools and interventions to support family caregivers. Journal of Telemedicine and Telecare.

[CR9] Council NR (2001). Educating Children with Autism.

[CR10] Ellison KS, Guidry J, Picou P, Adenuga P, Davis TE (2021). Telehealth and autism prior to and in the age of COVID-19: A systematic and critical review of the last decade. Clinical Child and Family Psychology Review.

[CR11] Esler AN, Bal VH, Guthrie W, Wetherby A, Weismer SE, Lord C (2015). The autism diagnostic observation schedule, toddler module: Standardized severity scores. Journal of Autism and Developmental Disorders.

[CR12] Ferguson J, Craig EA, Dounavi K (2018). Telehealth as a model for providing behaviour analytic interventions to individuals with autism spectrum disorder: A systematic review. Journal of Autism and Developmental Disorders.

[CR13] Gotham K, Pickles A, Lord C (2009). Standardizing ADOS scores for a measure of severity in autism spectrum disorders. Journal of Autism and Developmental Disorders.

[CR14] Hamad CD, Serna RW, Morrison L, Fleming R (2010). Extending the reach of early intervention training for practitioners. Infants & Young Children.

[CR15] Heitzman-Powell LS, Buzhardt J, Rusinko LC, Miller TM (2014). Formative evaluation of an aba outreach training program for parents of children with autism in remote areas. Focus on Autism and Other Developmental Disabilities.

[CR16] Hermaszewska S, Sin J (2021). End-user perspectives on the development of an online intervention for parents of children on the autism spectrum. Autism.

[CR17] Iadarola S, Levato L, Harrison B, Smith T, Lecavalier L, Johnson C, Swiezy N, Bearss K, Scahill L (2018). Teaching parents behavioral strategies for autism spectrum disorder (ASD): Effects on stress, strain, and competence. Journal of Autism and Developmental Disorders.

[CR18] Ingersoll B, Berger NI (2015). Parent engagement with a telehealth-based parent-mediated intervention program for children with autism spectrum disorders: Predictors of program use and parent outcomes. Journal of Medical Internet Research.

[CR19] Ingersoll B, Wainer AL, Berger NI, Pickard KE, Bonter N (2016). Comparison of a self-directed and therapist-assisted telehealth parent-mediated intervention for children with ASD: A pilot RCT. Journal of Autism and Developmental Disorders.

[CR20] Kaminski JW, Valle LA, Filene JH, Boyle CL (2008). A meta-analytic review of components associated with parent training program effectiveness. Journal of Abnormal Child Psychology.

[CR21] Keen D, Couzens D, Muspratt S, Rodger S (2010). The effects of a parent-focused intervention for children with a recent diagnosis of autism spectrum disorder on parenting stress and competence. Research in Autism Spectrum Disorders.

[CR22] Kim SH, Lord C (2011). New autism diagnostic interview-revised algorithms for toddlers and young preschoolers from 12 to 47 months of age. Journal of Autism and Developmental Disorders.

[CR23] Knutsen J, Wolfe A, Burke BL, Hepburn S, Lindgren S, Coury D (2016). A systematic review of telemedicine in autism spectrum disorders. Review Journal of Autism and Developmental Disorders.

[CR24] Koren PE, DeChillo N, Friesen BJ (1992). Measuring empowerment in families whose children have emotional disabilities: A brief questionnaire. Rehabilitation Psychology.

[CR25] Kuhn JC, Carter AS (2006). Maternal self-efficacy and associated parenting cognitions among mothers of children with autism. American Journal of Orthopsychiatry.

[CR26] Kuravackel GM, Ruble LA, Reese RJ, Ables AP, Rodgers AD, Toland MD (2017). COMPASS for hope: Evaluating the effectiveness of a parent training and support program for children with ASD. Journal of Autism and Developmental Disorders.

[CR27] Lindgren S, Wacker D, Suess A, Schieltz K, Pelzel K, Kopelman T, Lee J, Romani P, Waldron D (2016). Telehealth and autism: Treating challenging behavior at lower cost. PEDIATRICS.

[CR28] Lord C, Rutter M, Couteur AL (1994). Autism diagnostic interview-revised: A revised version of a diagnostic interview for caregivers of individuals with possible pervasive developmental disorders. Journal of Autism and Developmental Disorders.

[CR29] Lord C, Rutter M, DiLavore P, Risi S, Gotham K, Bishop S (2012). Autism diagnostic observation schedule-2nd edition (ados-2).

[CR30] Marchi FD, Contaldi E, Magistrelli L, Cantello R, Comi C, Mazzini L (2021). Telehealth in neurodegenerative diseases: Opportunities and challenges for patients and physicians. Brain Sciences.

[CR31] Moreland AD, Felton JW, Hanson RF, Jackson C, Dumas JE (2016). The relation between parenting stress, locus of control and child outcomes: Predictors of change in a parenting intervention. Journal of Child and Family Studies.

[CR32] Neely L, Rispoli M, Gerow S, Hong ER, Hagan-Burke S (2017). Fidelity outcomes for autism-focused interventionists coached via telepractice: A systematic literature review. Journal of Developmental and Physical Disabilities.

[CR33] Parsons D, Cordier R, Vaz S, Lee HC (2017). Parent-mediated intervention training delivered remotely for children with autism spectrum disorder living outside of urban areas: Systematic review. Journal of Medical Internet Research.

[CR34] Peter CCS, Brunson LY, Cook JE, Subramaniam S, Larson NA, Clingan M, Poe SG (2014). Adherence to discrete-trial instruction procedures by rural parents of children with autism. Behavioral Interventions.

[CR35] Pickard KE, Wainer AL, Bailey KM, Ingersoll BR (2016). A mixed-method evaluation of the feasibility and acceptability of a telehealth-based parent-mediated intervention for children with autism spectrum disorder. Autism.

[CR36] R Core Team (2018) R: A language and environment for statistical computing. R Foundation for Statistical Computing. https://www.R-project.org/

[CR37] Rogers SJ, Dawson G (2010). Early start Denver model for young children with autism: Promoting language, learning, and engagement.

[CR38] Rogers SJ, Dawson G, Vismara LA (2012). An early start for your child with autism: Using everyday activities to help kids connect, communicate, and learn.

[CR39] Simacek J, Elmquist M, Dimian AF, Reichle J (2020). Current trends in telehealth applications to deliver social communication interventions for young children with or at risk for autism spectrum disorder. Current Developmental Disorders Reports.

[CR40] Solomon, D., & Soares, N. (2020). Telehealth approaches to care coordination in autism spectrum disorder. In *Interprofessional care coordination for pediatric autism spectrum disorder*. Springer International Publishing, pp 289–306. 10.1007/978-3-030-46295-6_19

[CR41] Sparrow, S. S., Cicchetti, D., & Balla, D. A. (2005). Vineland adaptive behavior scales, second edition. 10.1037/t15164-000

[CR42] Symon JB (2001). Parent education for autism: Issues in providing services at a distance. Journal of Positive Behavior Interventions.

[CR43] Tonge B, Brereton A, Kiomall M, Mackinnon A, King N, Rinehart N (2006). Effects on parental mental health of an education and skills training program for parents of young children with autism: A randomized controlled trial. Journal of the American Academy of Child & Adolescent Psychiatry.

[CR44] Venkatesh, V., Morris, M. G., Davis, G. B., & Davis, F. D. (2003). User acceptance of information technology: Toward a unified view. *MIS Quarterly* pp. 425–478.

[CR45] Vismara LA, McCormick C, Young GS, Nadhan A, Monlux K (2013). Preliminary findings of a telehealth approach to parent training in autism. Journal of Autism and Developmental Disorders.

[CR46] Vismara LA, McCormick CEB, Wagner AL, Monlux K, Nadhan A, Young GS (2018). Telehealth parent training in the early start Denver model: Results from a randomized controlled study. Focus on Autism and Other Developmental Disabilities.

[CR47] Wainer AL, Ingersoll BR (2014). Increasing access to an ASD imitation intervention via a telehealth parent training program. Journal of Autism and Developmental Disorders.

[CR48] Wallisch A, Little L, Pope E, Dunn W (2019). Parent perspectives of an occupational therapy telehealth intervention. International Journal of Telerehabilitation.

[CR49] Weinstein RS, Lopez AM, Joseph BA, Erps KA, Holcomb M, Barker GP, Krupinski EA (2014). Telemedicine, telehealth, and mobile health applications that work: Opportunities and barriers. The American Journal of Medicine.

[CR50] Weiss JA, Robinson S, Fung S, Tint A, Chalmers P, Lunsky Y (2013). Family hardiness, social support, and self-efficacy in mothers of individuals with autism spectrum disorders. Research in Autism Spectrum Disorders.

